# Spontaneous and environment induced genomic alterations in yeast model

**DOI:** 10.1016/j.cellin.2024.100209

**Published:** 2024-09-26

**Authors:** Ke-Jing Li, Lei Qi, Ying-Xuan Zhu, Min He, Qian Xiang, Dao-Qiong Zheng

**Affiliations:** a State Key Laboratory (SKL) of Biobased Transportation Fuel Technology, Ocean College, Zhejiang University, Hangzhou, 316021, China; b Department of Molecular Genetics and Microbiology, Duke University, Durham, 27705, USA; c Lishui University, Lishui, 323000, China

**Keywords:** Spontaneous genomic alterations, Environment induced mutations, Yeast model, Adaptation, Whole-genome sequencing

## Abstract

While genomic alterations are fundamental to biological evolution, enabling adaptation and diversity, they can also result in detrimental outcomes, such as the development of genetic diseases including cancer. The budding yeast *Saccharomyces cerevisiae* serves as an exemplary model for investigating the mechanisms behind various genomic alterations, including point mutations, chromosomal rearrangements, and whole-chromosome aneuploidy. In this review, we highlight the application of genetic screening systems to assess the mutagenic effects of physical and chemical agents efficiently. Additionally, we discuss the utilization of high-throughput sequencing technologies to uncover comprehensive genomic alterations and rare genetic events. We provide a detailed summary of the features of genomic alterations and discuss the genetic mechanisms driving these changes under both spontaneous and stress-induced conditions. Given the high conservation of DNA replication and repair machinery across different organisms, the insights gained from studies on yeast offer valuable perspectives for understanding the delicate balance between genome plasticity and integrity in other species.

## 1 Introduction

Changes are inherent to both the world we live in and to organisms. Genomic alterations, or changes in DNA, can occur spontaneously or be induced by environmental factors. The budding yeast *Saccharomyces cerevisiae* has emerged as a versatile and robust model system for studying genome evolution due to its well-characterized genetic background and ease of manipulation (Botstein et al., 1997). Like other organisms, the frequency of spontaneous genomic alterations in yeast is low (Hall et al., 2008; Lynch et al., 2008) due to the presence of sophisticated DNA repair systems, including base excision repair (BER), nucleotide excision repair (NER), mismatch repair (MMR), non-homologous end joining (NHEJ), and homologous recombination (HR). However, under stressed conditions such as ultraviolet (UV) (Yin et al., 2017; Yin & Petes, 2013), bleomycin (Zheng et al., 2022), furfural (Qi et al., 2019; Qi et al., 2023b), high concentration of sodium (Liu & Zhang, 2019), and heat shock (Shen et al., 2020b), the repair capacity of yeast cells may be overwhelmed, leading to the accumulation of unrepaired DNA lesions and increased mutation rates.

In this review, we define genomic alterations to include point mutations [single nucleotide variations (SNVs) and small insertions and deletions (InDels)], loss of heterozygosity (LOH), chromosomal rearrangements (large deletions, duplications, inversions, and translocations), and whole-chromosome aneuploidy. By analyzing the frequency and spectrum of these genetic events, researchers can infer the types of DNA damage caused by exogenous agents and understand the repair pathways involved (Alexandrov et al., 2020; Thatikonda et al., 2023). This knowledge can help assess the risk of genetic diseases associated with exposure to these exogenous agents (Brady et al., 2022; Helleday et al., 2014). Traditionally, reporter gene-based genetic screening systems were widely used to evaluate the mutagenesis of physical and chemical agents. In recent years, the growing application of whole-genome sequencing in yeast genetics has further enriched our understanding of how a eukaryotic genome evolves in response to environmental changes. Below, we explore the strategies developed using the yeast model to investigate the patterns of spontaneous and environment-induced genomic alterations. We delve into the underlying genetic mechanisms driving these changes, highlighting how yeast has served as a powerful system to elucidate the complexities of genome evolution and mutation dynamics. By examining both spontaneous and stress-induced genetic variations, we aim to provide a comprehensive understanding of how genomes adapt and respond to environmental challenges.

## 2 Experimental approaches for studying genomic alterations in yeast

### 2.1 Forward and reverse genetic screening systems

**Fig. 1:**
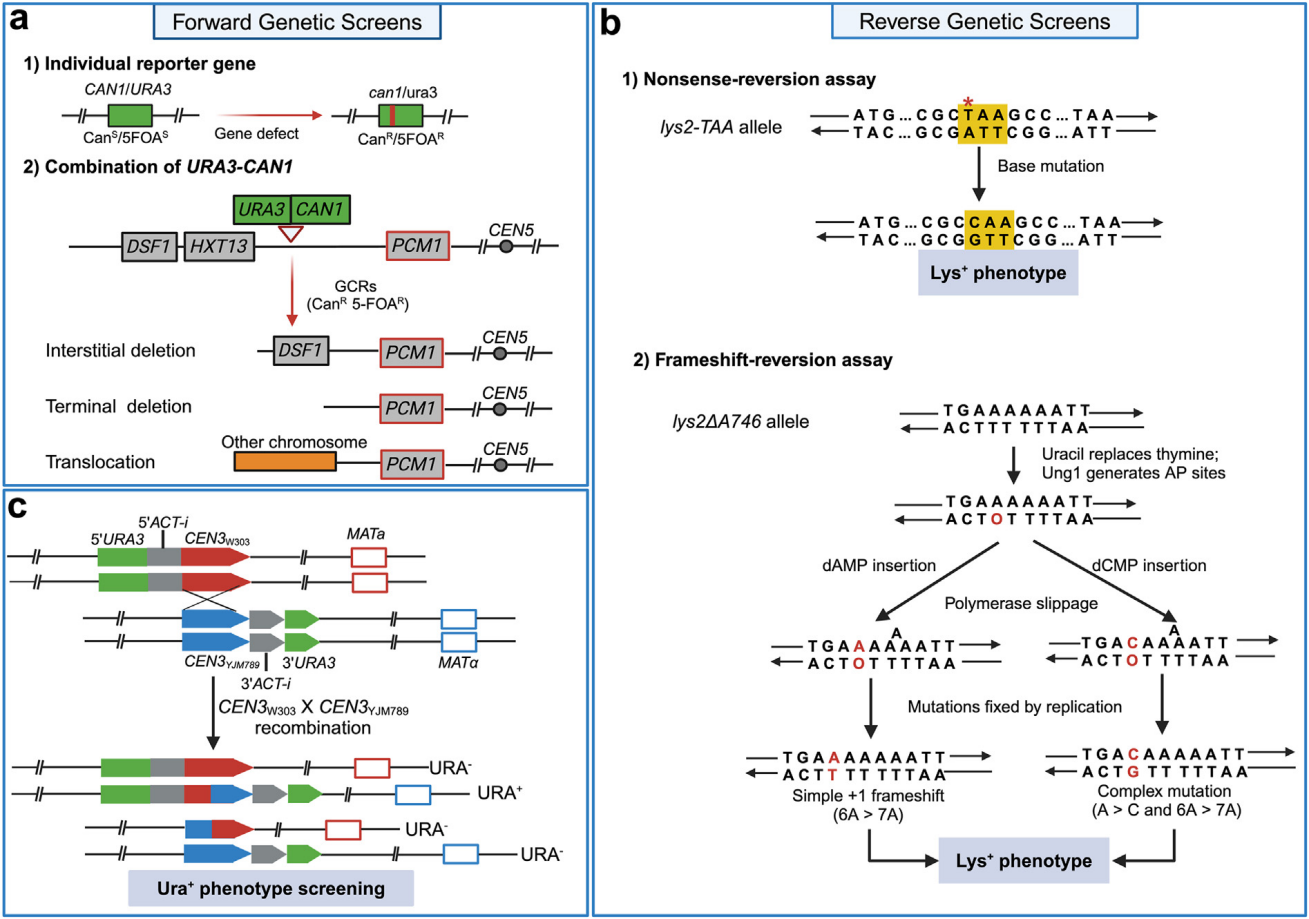
**Forward and reverse genetic screens.** a Forward genetic screening using report genes *CAN1* and *URA*3. Mutations are detected by selecting mutants that acquire resistance to canavanine or 5-FOA. The *CAN1* and *URA3* cassettes were inserted near the telomeric region adjacent to the essential gene *PCM1*, within a region of non-essential genes. Various gross chromosomal rearrangements, including interstitial deletions, terminal deletions, and translocations, can be selected based on resistance to both canavanine and 5-FOA [modified from Srivatsan et al. (2018)]. b Reverse genetic screening with nonsense- and frameshift-reversion assays: In the nonsense-reversion assay, a TAA stop codon is inserted upstream of the *LYS2* gene's termination codon, creating the *lys2*-TAA allele. Mutations that convert this TAA codon into a non-termination codon restore the Lys^+^ phenotype. In the frameshift-reversion assay, uracil replaces one of the thymines opposite the 6A run, generating an AP site. Bypassing this site by inserting dAMP or dCMP, followed by slippage, results in either a straightforward 6A–7A frameshift or a complex frameshift involving an A to C transversion. Mutations in either assay can restore the function of the *LYS2* gene, allowing growth on plates lacking lysine [modified from Kim et al. (2011)]. c Method for detecting recombination between centromeres. Chromosome III homologs derived from W303 and YJM789 are depicted in red and blue, respectively. On the W303-derived homolog, *CEN3*_w303_ is located next to the 5′ end of the *ACT1* intron, which is inserted downstream of the 5′ portion of *URA3*. In contrast, the YJM789-derived homolog has *CEN3*_YJM789_ adjacent to the 3′ end of the *ACT1* intron, positioned next to the 3′ end of *URA3*. A reciprocal crossover within the centromeric sequences would produce an intact *ACT1* intron containing a recombinant centromere within a complete *URA3* gene [modified from Kozmin et al. (2024)].

Linking microscopic DNA mutations with observable phenotypes enables efficient study of genetic variation in yeast. Forward and reverse genetic screening leverage the loss or restoration of specific genes to produce detectable phenotypic changes in cells. This approach allows researchers to identify DNA mutations based on their impact on observable traits (Fig. 1a). For forward screening, genes such as *CAN1* and *URA3* are utilized as reporters. Loss of function in these genes results in resistance to canavanine and 5-fluoroorotic acid (5-FOA), respectively. By plating yeast cells on media containing canavanine or 5-FOA and counting the resultant colonies, the forward mutation frequency of *CAN1* or *URA3* can be quantified (Ekwall & Ruusala, 1991). Subsequent PCR amplification and sequencing of the mutated alleles of *ura3* or *can1* reveal the DNA mutation spectrum (Lang & Murray, 2008; Zheng et al., 2022). Additionally, when *CAN1* and *URA3* are tandemly inserted at a specific chromosomal location, yeast colonies resistant to both canavanine and 5-FOA can be screened to identify chromosomal rearrangements, including interstitial deletions, terminal deletions, and translocations (Fig. 1a) (Srivatsan et al., 2018).

While various reverse screening reporters have been used in yeast genetics, the *LYS2* gene serves as a notable example. In the nonsense-reversion assay, a TAA stop codon is introduced before the termination codon of the *LYS2* gene, creating the *lys2-TAA* allele. If this TAA codon mutates to a non-termination codon, a Lys^+^ phenotype is selectable (Fig. 1b) (Greene & Jinks-Robertson, 1997; Harfe & Jinks-Robertson, 1999, 2000; Kim et al., 2011). Additionally, the *LYS2* gene has been modified for frameshift-reversion screening. The *lys2-ΔA746* allele, where a 7A run is reduced to a 6A run, allows for the selection of +1 frameshifts (Fig. 1b) (Kim et al., 2011). This system was also used to explore which deoxynucleotide monophosphates (dNMPs) are preferentially incorporated opposite apurinic/apyrimidinic (AP) sites by analyzing base substitutions within the 6A run. When thymine is replaced by uracil, the Ung1 enzyme identifies and removes the uracil, creating an AP site opposite the 6A run. A to C transversions were found to be the most frequent base substitutions, often accompanied by +1 frameshifts in Lys^+^ mutants. The findings indicated that deoxycytidine monophosphate (dCMP) is the most frequently inserted dNMP opposite the AP site, with this insertion being entirely dependent on the catalytic activity of Rev1 (Kim et al., 2011). Reverse screening systems are not only effective for monitoring point mutations but also valuable for studying other genetic events, such as homologous recombination. For example, a recent study by Kozmin et al. (2024) developed a system to detect centromere-centromere recombination using the principle of *URA3* reversion mutation. In heterozygous diploid yeast, the 5′ end of *URA3* and the 5′ portion of an *ACT1* intron is positioned adjacent to the centromere on the W303-derived chromosome III. Conversely, the 3′ end of *URA3* and the 3′ portion of the *ACT1* intron is positioned adjacent to the YJM789 centromere (Fig. 1c). Recombination between these centromeres results in the generation of a wild-type *URA3* gene that contains the *ACT1* intron. By calculating the frequency of Ura ^+^ mutants, the centromere-centromere recombination frequency can be estimated at approximately 10⁻⁸ per cell division (Kozmin et al., 2024).

### 2.2 Sectored colonies screening system

**Fig. 2:**
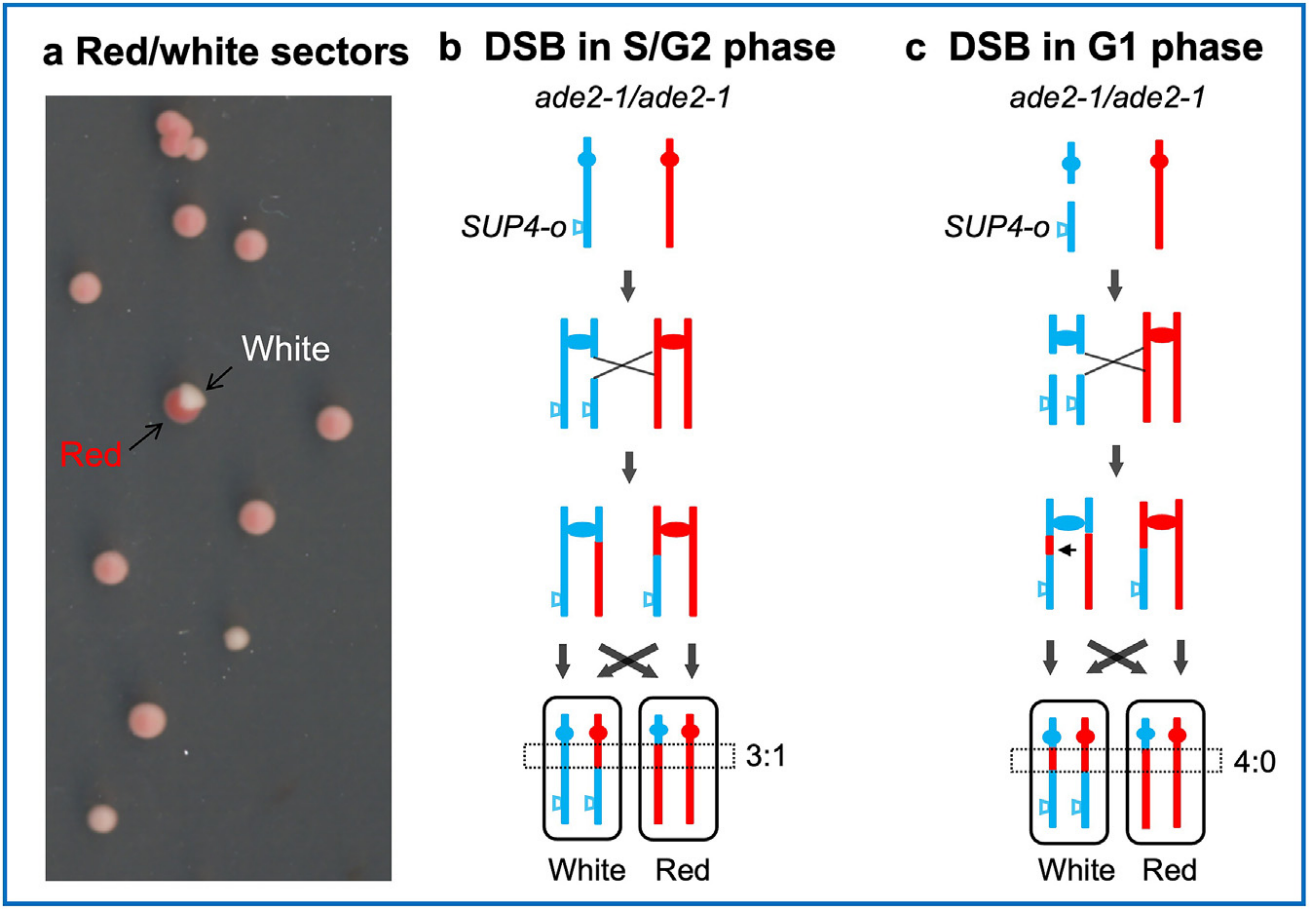
**White/red sectoring colony screening system.** a Red/white sectors on plates. b-c DSBs occurring in different phases of the cell cycle. Red and blue lines represent homologous chromosomes in a diploid yeast strain homozygous for the *ade2-1* allele. One copy of the *SUP4*-o gene is inserted at the end of one homolog. This insertion partially suppresses the ochre mutation of *ade2-1*, leading to the appearance of pink colonies. b Reciprocal crossover between homologous chromosomes during the S/G2 phase can lead to one daughter cell inheriting two copies of the *SUP4*-o gene, resulting in a white phenotype, while the other daughter cell, lacking *SUP4*-o, appears red. This process involves repair of a double-strand break (DSB) and results in a 3:1 gene conversion tract associated with the crossover event. c A DSB occurring in the G1 phase on one homolog leads to both sister chromatids harboring DSBs. Repairing these broken chromatids in the S/G2 phase can produce gene conversion tracts with 4:0 regions, where all four chromatids, including the repaired ones, exhibit identical single nucleotide polymorphisms (SNPs).

*ADE2* gene is essential for adenine biosynthesis in yeast (Dorfman, 1969). An ochre mutation at codon 65 (*ade2-1*), introducing a premature stop codon (UAA), causes cells to accumulate red pigment (Hawthorne, 1969). The *SUP4* gene in yeast encodes tRNA-Tyr, and its mutant form, *SUP4*-o, can recognize the UAA codon and insert tyrosine, allowing the continuation of Ade2 peptide chain synthesis (Kurjan et al., 1980). In diploids homozygous for the *ade2-1* mutation and possessing zero, one, or two copies of *SUP4*-o, red, pink, and white colonies are observed, respectively (Barbera & Petes, 2006). As shown in Fig. 2, when a single copy of the *SUP4*-o gene is inserted near the end of one homologous chromosome in a diploid, homologous recombination between the centromere and *SUP4*-o during mitosis can result in one daughter cell losing *SUP4*-o and the other gaining two copies of *SUP4*-o. This can completely suppress the *ade2-1* mutation and prevent red pigment accumulation. Consequently, if chromosomal exchange occurs during the first cell division after plating this diploid yeast, red and white sectored colonies will form (Fig. 2a). Charles and Petes (2013) utilized this system in strain JSC25-1, inserting *SUP4*-o at the end of YJM789-derived chromosome IV, observing sectored colonies at a frequency of approximately 3.6 × 10⁻⁵.

The sectored colony screening not only quantitatively analyzes reciprocal crossovers but also identifies the timing (cell cycle phase) and location (chromosomal region) of DNA double-strand breaks (DSBs) that initiate the crossover. Using SNP microarrays designed with W303 and YJM789 strain information, researchers can analyze the gene conversion tracts associated the crossover in sectored colonies (Charles & Petes, 2013; St Charles et al., 2012). If a DSB occurs in one sister chromatid during the S/G2 phase and the homologous chromosome is used as a template for repair, the resulting gene conversion tract will exhibit a 3:1 SNP pattern (Fig. 2b). Conversely, if a DSB occurs during the G1 phase, leading to breaks on both sister chromatids, the gene conversion tract will show a 4:0 SNP pattern (Fig. 2c). Previous studies employing this system have explored the effects of various stressors, such as heat shock, Zeocin, hydrogen peroxide (H_2_O_2_), and furfural, on the induction of DSBs in the G1 phase (Qi et al., 2019; Qi et al., 2023a; Shen et al., 2020b; Yin et al., 2017; Yin & Petes, 2013; Zhang et al., 2019; Zheng et al., 2022). These investigations revealed that the distribution of crossover breakpoints differs between spontaneous and stress-induced conditions, indicating that specific stressors can induce unique chromosomal fragile sites.

### 2.3 High-throughput sequencing for detecting whole genome variations

Mutations are the primary source of genetic evolution. Under natural conditions, genomic mutations occur infrequently, making mutation accumulation (MA) an essential process (Drake et al., 1998). For a considerable period, direct estimation of mutation rates was mainly limited to the analysis of a few phenotypic reporter genes (Drake et al., 1998; Huang et al., 2003; Smith et al., 2004). With the advancement of high-throughput technology, a new and promising approach to studying mutation rates involves combining MA experiments with whole-genome sequencing (Denver et al., 2009; Keightley et al., 2009; Lynch, 2010; Lynch et al., 2008; Ossowski et al., 2010). Whole-genome sequencing enables the detection of SNVs and small InDels that microarray cannot detect. Zhu et al. (2014) conducted continuous passage MA experiments on 145 diploid yeast strains and sequenced them using the Illumina platform. They found that the spontaneous frequencies of SNVs and InDels per base per cell division in diploid yeast strains were 1.7 × 10^−10^ and 5.0 × 10^−12^, respectively. Besides SNVs and InDels, it can also identify LOH events in heterozygous yeast, similar to microarrays but with higher accuracy (Sui et al., 2020).

The short reads of the next-generation high-throughput sequencing technologies, notably the Illumina and BGI platforms, pose challenges in the detection of complex chromosomal structural variation events, especially those involving long repeat sequences. Recent years, third-generation single-molecule sequencing technologies represented by Pacific Biosciences (PacBio) and Oxford Nanopore Technologies (ONT) have enabled reads to reach tens of kb or even Mb, providing efficient solutions for de novo genome assembly and extensive structural variation detection (Rhoads & Au, 2015; Wang et al., 2021). Yue et al. (2017) examined evolutionary genome dynamics through structural rearrangements in 12 domesticated and wild yeast strains. The significant differences in rearrangements between wild and domesticated yeasts highlighted the impact of human activities on genomic structure genome evolution. O'Donnell et al. (2023) used ONT technology to sequence and assemble 142 *S. cerevisiae* strains, uncovering 4800 chromosomal structural variations. These variations provided a comprehensive view of the genome's structural landscape in *S. cerevisiae*, enhancing our understanding of genome evolution at a population level. Additionally, tetrad sequencing of budding yeast using RecombineX enables the detection of chromosomal rearrangements specifically induced by meiosis (Li et al., 2022).

Specific regions of the genome can also be detected. Jiang et al. (2024) tackled the challenge of studying ribosomal DNA (rDNA)'s repetitive and dynamic structure by leveraging the loxPsym site and Cre recombinase in yeast to create a system with variable rDNA copy numbers. Employing ONT sequencing, they observed changes in rDNA copy number and its effects on yeast. Remarkably, the study found that rDNA copy number could be reduced to as low as eight copies without compromising nucleolus formation, cell growth, or transcriptome profiles. The repetitive nature of telomeres hinders next-generation sequencing in resolving allele-specific compositions of chromosomal arms and individual telomeres. Sholes et al. (2022) introduced a technique for tagging telomeres by annealing a complementary oligo(dA) sequence to the poly(T) sequence at the TeloTag oligonucleotide end, which contains a unique TeloTag sequence. This method allowed them to measure telomere length through nanopore sequencing in *S. cerevisiae*. Their study revealed that chromosome end-specific telomere lengths remained stable over 120 cell divisions. Additionally, sequencing telomeres in a telomerase-null mutant (*est2Δ*) identified a minimal telomere length of approximately 75 bp. Recently, researchers have been exploring the dynamic mechanisms of telomere sequences in aging and cancer cells. Schmidt et al. (2024) used high-resolution long-read telomere sequencing to differentiate between bulk, chromosome arm-specific, and allele-specific telomere lengths in humans. This approach allowed for the distinction between telomerase-positive and alternative lengthening of telomeres (ALT)-positive cancer cell lines.

Overall, the long reads provided by third-generation sequencing platforms make them powerful tools for analyzing genome instability, particularly in the context of large-scale chromosomal rearrangements, rDNA copy number variations (CNVs) and telomere length changes. However, a limitation of ONT is its lower accuracy in base calling and detection of small mutations, despite its ability to produce the longest reads. Integrating data from both next-generation and third-generation sequencing technologies enables a more comprehensive analysis of genomic variations.

## 3 Spontaneous genomic alterations in *S. cerevisiae*

### 3.1 SNVs and InDels

**Fig. 3:**
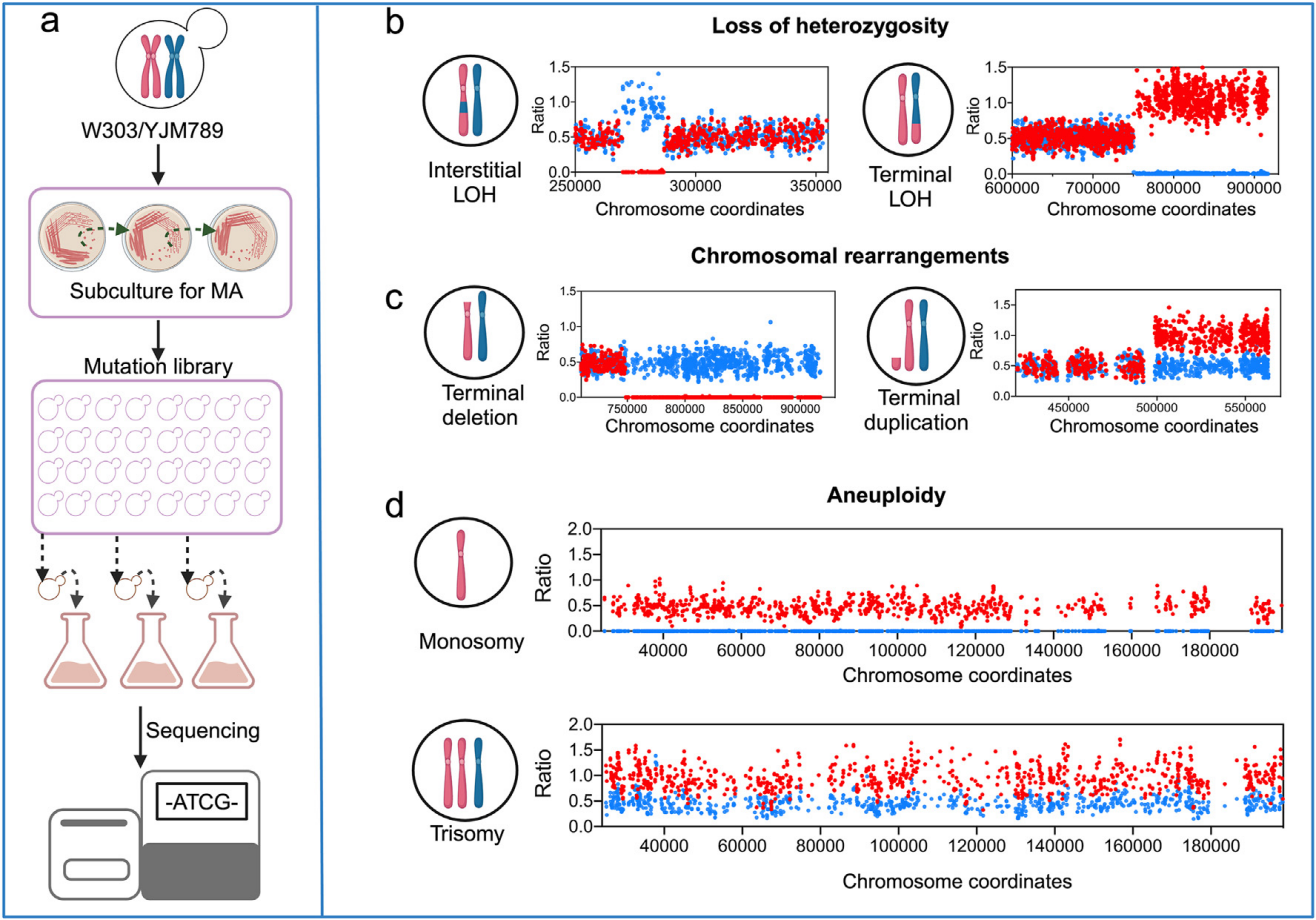
**Mutation accumulation andwhole-genomeanalysis of genomic alterations.** a. Experimental workflow for mutation accumulation of diploid yeast cells and next-generations sequencing. Panels b–d present the genomic alterations detected through genome sequencing. The blue and red points denote SNPs between homologous chromosomes. Sequencing coverage values of 0, 0.5, and 1 correspond to zero, one, and two copies of SNPs, respectively. b. Loss of heterozygosity (LOH) include interstitial LOH (left) and terminal LOH (right). c. Chromosomal rearrangements: terminal deletions (left) and terminal duplication (right). d. Whole-chromosome aneuploidy events: monosomy (above) and trisomy (below).

Early studies of forward mutation rates in the *URA3* and *CAN1* genes found mutation rates about 4 × 10^−10^ and 6 × 10^−10^/base/cell division, respectively (Lang & Murray, 2008). Sui et al. (2020) conducted an experiment in which 93 MA lines derived from a wild-type diploid *S. cerevisiae* were sub-cultured independently (Fig. 3a). The *S. cerevisiae* isolates in this experiment underwent a total of 264,000 cell divisions, accumulating thousands of genomic alterations. They detected 1265 SNVs, indicating the spontaneous rate of SNVs on *S. cerevisiae* genome was 2.10 × 10^−10^/base/cell division. This result was consistent with that reported in the other two MA experiment by Zhu et al. (2014) (1.67 × 10^−10^/per base/cell division) and Nathaniel et al. (2018) (2.89 × 10^−10^/per base/cell division). In the absence of selective pressure, the SNVs rate correlated positively with chromosome size and was relatively evenly distributed across chromosomes, although some reports indicated that CpG island sequences exhibit higher mutation rates (Capuano et al., 2014; Sharp et al., 2018). The ratio of transitions to transversions showed no significant difference across different strain backgrounds, ranging between 0.7 and 0.9 (Melde et al., 2022; Sharp et al., 2018; Zhu et al., 2014). The rate of SNVs is not influenced by the mating type in haploids. However, in the same experiment, haploid strains exhibited an approximately 40% higher rate of SNVs compared to diploid strains (Sharp et al., 2018). We recently evaluated the SNVs rate in a *rev3* mutant with the same background as the strain used in the study by Sui et al. (2020). Our unpublished data showed that the deletion of the *REV3* gene (encoding the catalytic subunit of DNA polymerase ζ) led to a 35% decrease in the SNVs rate, indicating that approximately one-third of spontaneous mutations in wild-type yeast are due to the activity of the error-prone DNA polymerase ζ.

The frequency of InDels is approximately an order of magnitude lower than that of SNVs, with a rate of 1.67× 10^−11^/base/cell division (Sui et al., 2020). Most spontaneous InDels occur in mononucleotide and dinucleotide repeat tracts, suggesting that they are likely caused by DNA polymerase slippage (Sui et al., 2020). Additionally, up to 18% of InDels are attributed to short-repeat (4–13 bp) mediated DNA sequence pop-out events exceeding 10 bp. The rarest genetic event observed was short palindromic sequence-mediated short inversions, likely due to template switching during DNA replication, with a frequency of approximately 4.94 × 10^−13^/base/cell division (Sui et al., 2020). Although template slippage and switching are relatively infrequent under normal conditions, these mechanisms can be significantly amplified by DNA replication stress (Abraham & Hazkani-Covo, 2021; Giannattasio et al., 2014), a characteristic often observed in tumor cells.

### 3.2 LOH

Numerous studies have underscored the significant role of LOH in somatic mosaicism, which leads to genetic alterations with notable phenotypic consequences, including those affecting cancer development (Nomura, 2020; O'Keefe et al., 2010). In the context of spontaneous genomic alterations, LOH events constitute approximately 50% of the total events observed in wild-type diploid *S. cerevisiae* (Sui et al., 2020). LOH events are categorized into two types based on whether the conversion tract extends to the chromosome end: interstitial LOH (I-LOH) and terminal LOH (T-LOH) (Fig. 3b), with observed frequencies of 3.3 × 10^−3^ and 1.4 × 10^−3^/cell division, respectively (Sui et al., 2020). Both I-LOH and T-LOH generally correlate with chromosome size, though these frequencies can vary based on genetic background. For instance, Abhishek et al. reported that LOH frequency increases with higher ploidy levels, with rates of 9.3 × 10^−3^, 22 × 10^−3^, and 84 × 10^−3^/cell division in diploids, triploids, and tetraploids, respectively (Dutta et al., 2022). Additionally, Sampaio et al. (2020) observed that *S. cerevisiae* strains carrying a LOH event at a specific chromosome site often exhibited multiple unselected rearrangements elsewhere in their genome. Their findings suggest that a subset of mitotic cells can undergo episodes of systemic genomic instability, during which the entire genome becomes susceptible to multiple genomic alterations within a short time frame.

During mitotic growth in yeast, most LOH events are initiated by DSBs that are repaired through homologous recombination. Sui et al. (2020) found that these LOH events, when mapped onto chromosomes, exhibit non-random distribution patterns. Specifically, breakpoints associated with I-LOH were predominantly located near centromeres, whereas T-LOH breakpoints were enriched close to telomeres (within 20 kb of the chromosome ends) (Sui et al., 2020). The distinct distributions of I-LOH and T-LOH breakpoints suggest that cells preferentially use different homologous recombination pathways depending on the location of DSBs. Moreover, the I-LOH and T-LOH breakpoints are associated with specific sequence elements. For example, I-LOH breakpoints are commonly found in replication termination zones and regions with low GC content. In contrast, T-LOH breakpoints are enriched in G4 quadruplex sequences, areas with high levels of γH_2_AX, and noncoding RNA genes (Sui et al., 2020). These observations highlight that the distribution of LOH breakpoints is influenced by both the spatial context of DSBs and the specific genomic features present at these sites. Finally, LOH events in yeast populations are also influenced by selective pressure. Several studies have demonstrated that T-LOH events occurring on the right arm of chromosome IV, which result in the conversion of the heterozygous *ssd1/SSD1* allele to the homozygous *SSD1/SSD1* allele, contribute to enhanced resistance to furan-derived compounds and high temperatures. These beneficial T-LOH are strongly selected for under such stressful conditions (Qi et al., 2019; Qi et al., 2023b; Zheng et al., 2022; Zhu et al., 2024).

### 3.3 Gross chromosomal rearrangements and aneuploidy

In addition to LOH, large-scale chromosomal rearrangements (such as amplifications or deletions greater than 1 kb) and the gain or loss of whole chromosomes are also prevalent in human cells. These alterations are often linked to a variety of diseases (Collins et al., 2020; Perry et al., 2008; Weckselblatt & Rudd, 2015; Weischenfeldt et al., 2013). Recent analyses of extensive whole-genome sequencing datasets have provided valuable insights into the characteristics of structural variations and their cross-genomic impacts across different types of cancers (Dubois et al., 2022). Unlike LOH, which primarily involves changes in allele frequencies, these genetic events result in copy number alterations of abundant genes. In contrast to the MA experiments conducted by Sharp et al. (2018) and Zhu et al. (2014), Sui et al. (2020) identified a greater frequency of large duplications and deletions (Fig. 3c). Their study provided more precise estimates for these events, with rates of 4.6 × 10^−5^ and 13.4 × 10^−5^/cell division, respectively. These chromosomal rearrangements are frequently observed in regions containing repetitive sequences, such as Ty elements and long terminal repeats (LTRs) (Sui et al., 2020), indicating they were resulted from homologous recombination between non-allelic repeat sequences. Qi et al. (2023a) and Fleiss et al. (2019) demonstrated that ectopic recombination between Ty elements is a significant source of structural variations.

For wild-type *S. cerevisiae* cells, aneuploidy (Fig. 3d) frequencies have been observed to range from 6 to 11 × 10^−5^ per cell division, with a notable predominance of trisomy events compared to monosomy and tetrasomy (Sharp et al., 2018; Sui et al., 2020; Zhu et al., 2014). While the process of non-disjunction theoretically leads to equal frequencies of monosomy and trisomy, monosomy has more severe negative effects on yeast viability. As a result, daughter cells with monosomy events are less likely to be selected. Furthermore, similar to LOH, aneuploidy occurs more frequently in tetraploids than in diploids and triploids (Dutta et al., 2022). Previous studies have demonstrated that aneuploidy can affect all 16 chromosomes of yeast, though the likelihood of such events varies significantly among them. Smaller chromosomes, such as I, III, and IX, are particularly prone to loss (Sui et al., 2020). While there is evidence suggesting a slight negative correlation between chromosome size and aneuploidy frequency, chromosome size alone does not fully explain the variation in aneuploidy rates. Despite being generally detrimental to cell division and development under normal conditions (Dutta et al., 2022), aneuploidy can offer cells a mechanism for rapid adaptation to environmental changes, potentially conferring evolutionary advantages (Replogle et al., 2020; Sheltzer & Amon, 2011; Zhu et al., 2018).

While geneticists often emphasize the genetic diversity arising from sexual reproduction, recent studies have underscored the crucial role that mitotic genome alterations play in generating genetic diversity. These findings illuminate the relative frequencies of various genomic alterations across eukaryotic genomes and raise new questions, such as the reasons behind the specific distribution patterns of I-LOH and T-LOH in relation to chromosome context.

## 4 Environmental factors induced genomic alterations in yeast

### 4.1 Radiation

**Fig. 4:**
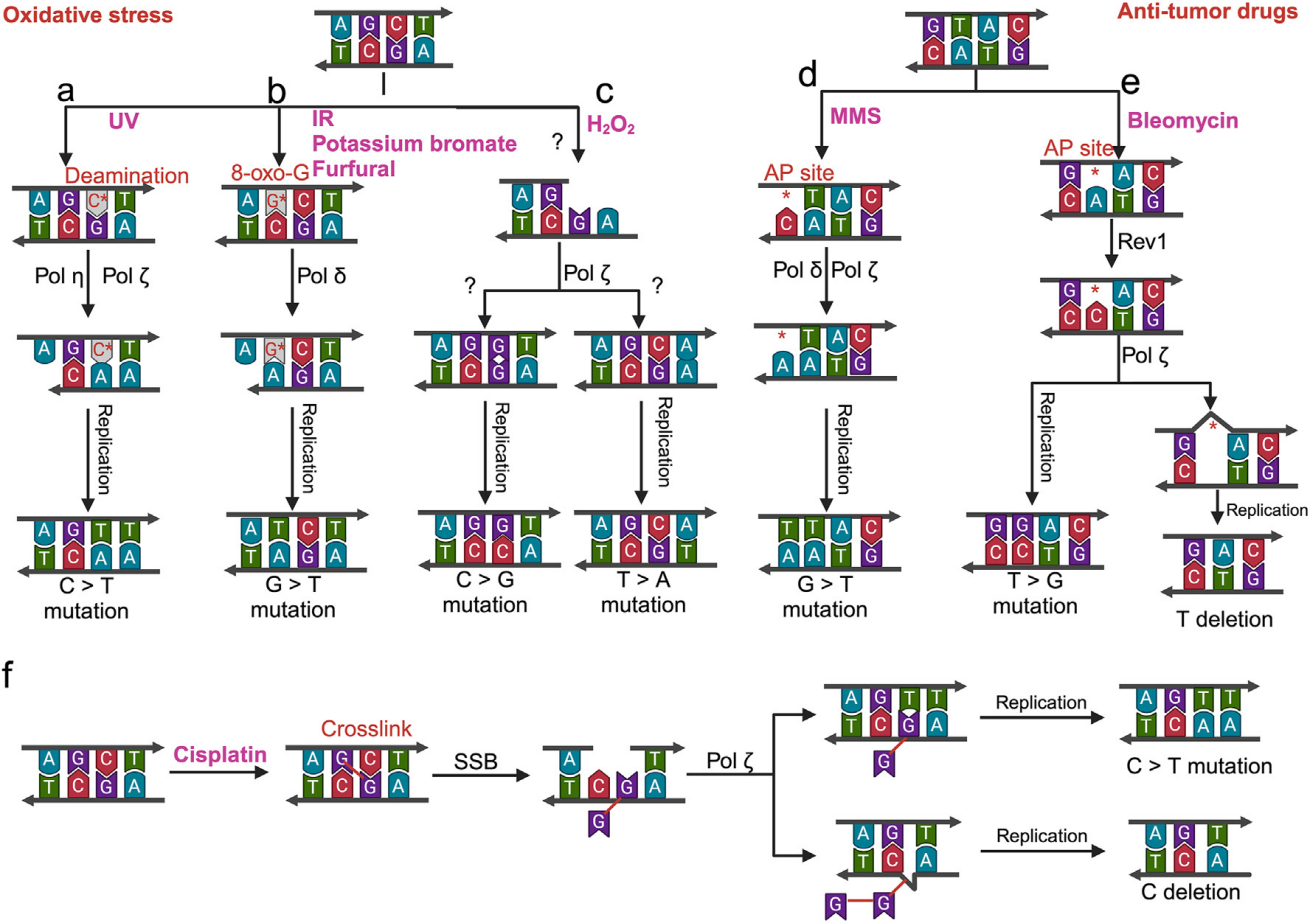
**Mutation spectra and possible genetic mechanisms.** Factors a-c are related to oxidative stress, and d-f are antitumor drugs. a. UV radiation causes damage at dipyrimidine sites. Cytosine deamination leads to the insertion of adenine opposite the deaminated cytosine by DNA polymerase ζ or η, resulting in C > T substitutions. b. IR, potassium bromate, and furfural induce reactive oxygen species (ROS) that oxidize guanine to 8-oxo-G. This oxidized base pairs with adenine, causing G > T mutations. c. Under H_2_O_2_ treatment, there is a significant increase in C > G or A > T mutations, likely due to the involvement of DNA polymerase ζ in DNA repair and replication. d. During MMS treatment, guanine bases are prone to depurination, forming apurinic/apyrimidinic (AP) sites. Adenine is preferentially inserted opposite these sites, leading to G > T mutations upon repair and replication. e. Bleomycin treatment causes frequent AP sites at 5′-GT-3′ motifs. Rev1 recognizes AP sites and preferentially inserts cytosine, resulting in T > G mutations. f. Cisplatin treatment induces crosslinks leading to single-strand breaks. During the repair process, DNA polymerase ζ may cause C > T substitutions or C deletions.

UV radiation is a powerful mutagen primarily originating from the sun and is the major cause of human skin cancer (Cleaver, 2002; Narayanan et al., 2010). Long-wave UVA (320–400 nm) causes indirect DNA damage through oxidative stress (Kozmin et al., 2005), whereas UVB (290–320 nm) and UVC (100–290 nm) directly induce covalent bonds between adjacent pyrimidines (Ravanat et al., 2001). Using the forward mutation assay of the *URA3* gene in *S. cerevisiae* to detect UV-induced base alterations, Lee et al. (1988) found that 5′-TT-3′ and 5′-CT-3′ sites are common targets for UV-induced SNVs in stationary phase yeast cells. To verify more general and representative UV signature mutations, Brash (2015) compiled and tested mutation datasets from cells exposed to UVA, UVB, UVC, or solar simulator light. UV-induced mutations are distinctively characterized by a prevalence of ≥60% C > T transitions at dipyrimidine sites, accompanied by ≥ 5% CC > TT transitions, underscoring the mutagenicity of pyrimidine dimers (Alexandrov et al., 2020; Brash, 2015; Shen et al., 2020a). The primary UV-generated photoproducts, cyclobutane pyrimidine dimers (CPDs) and 6-4 pyrimidine-pyrimidone photoproducts (6-4 PPs), pose significant obstacles to replicative polymerases. Translesion synthesis (TLS) polymerases mitigate replication blockages, albeit introducing mutations in the process (Maiorano et al., 2021; Waters et al., 2009). While error-prone DNA polymerases were once thought to be the primary culprit for these mutations (Abdulovic & Jinks-Robertson, 2006; Siebler et al., 2022), recent research suggests that DNA polymerase η′s accurate replication of CPDs may be the key factor. In this intricate mechanism, CPDs serve as templates for Polymerase η, facilitating the deamination of C (to U) within the dimers, which then guides the correct insertion of A opposite the deaminated sites, leading to C > T transitions (Fig. 4a) (Menck et al., 2024). This is evidenced by Polymerase η′s precise bypass of TT CPDs in cell extracts, where AA is accurately inserted opposite the lesions (Washington et al., 2000; Yoon et al., 2009). Beyond point mutations, UV exposure also triggers great recombination events. Yin and Petes (2013) leveraged SNP microarrays to conduct genome-wide, high-resolution mapping of UV-induced mitotic recombination in *S. cerevisiae*. Notably, even at UV doses that do not severely compromise cell survival (>70%), the frequency of LOH soars, increasing thousands of times compared to untreated cells. Moreover, high UV doses (15 J/m^2^) elicit a greater number of DSBs than lower doses, further illustrating the profound impact of UV radiation on genomic integrity and stability.

Ionizing radiation (IR) is a potent environmental mutagen and carcinogen that can inflict both direct and indirect damage to DNA. The direct effects of IR involve the direct deposition of energy within DNA molecules, resulting in various types of lesions, including base lesions and DNA breaks (Lomax et al., 2013). The frequency of these lesions increases with the amount of radiation exposure, as evidenced by the number of lesions induced per cell per Gray (Gy) of radiation. For example, under low linear energy transfer (LET) γ-radiation, ionizing radiation induces approximately 850 pyrimidine lesions, 450 purine lesions, 1000 single-strand breaks (SSBs), and 20–40 DSBs per cell per Gy (Cadet et al., 2008). In addition to direct DNA damage, IR also induces the production of intracellular reactive oxygen species (ROS) (Leach et al., 2001; Lomax et al., 2013), which can cause further DNA damage through oxidative stress (described below) (Waris & Ahsan, 2006). Moreover, IR can cause chromosome aberrations (CAs) through homologous recombination between non-allelic repetitive elements, particularly Ty retrotransposons. These repeat-associated DSBs can lead to chromosomal rearrangements and reshape the genome, potentially driving evolutionary change (Argueso et al., 2008). Recent studies using large-scale whole-genome sequencing of irradiated mouse and human single-cell amplified clones have provided further insights into the mutational burden induced by IR. These studies have shown that, despite high intercellular randomness, an average of 2.33 mutational events/Mb of the genome occur following 1 Gy of IR exposure (Youk et al., 2024). Importantly, the study found that the mutation burden depends primarily on the total irradiation dose, rather than the dose rate or cell type (Youk et al., 2024).

### 4.2 Extreme temperature

Generally, the optimal growth temperature for yeast is 25–35 °C, with deviations from this range accelerating mutation rates (Ogur et al., 1960). An investigation by Shen et al. (2020b) highlights the profound impact of extreme heat shock, specifically 52 °C exposure for brief periods, on yeast cells. This treatment resulted in a remarkable nearly tenfold surge in recombination frequency and a staggering 100-fold increase in the occurrence of chromosomal aberrations, encompassing both rearrangements and whole chromosome amplifications (Shen et al., 2020b). Furthermore, Gusa et al. (2023) advanced the hypothesis that under heat stress conditions, the human fungal pathogen *Cryptococcus neoformans* utilizes transposon mutagenesis as a primary mechanism to induce spontaneous mutations. Their investigation revealed a substantial accumulation of transposable element (TE) copies within the genomes of transposon accumulation (TA) lines that were propagated at the host-relevant temperature of 37 °C, in contrast to those maintained at 30 °C. This marked increase in TE copy number at 37 °C was attributed to the heightened mobility of the retroelements Tcn12 and Cnl1, which belong to the LINE-1 family in *C. neoformans*. Notably, the rate of TE mutation at 37 °C exceeded the combined rate of SNVs and InDels by a factor of three, emphasizing that TE mutations, rather than minor sequence variations, are the primary drivers of spontaneous genomic alterations in *C. neoformans* under heat stress conditions (Gusa et al., 2023).

On one hand, as demonstrated above, high temperatures can stimulate genomic alterations to accelerate phenotypic evolution. However, Zheng et al. (2024) provide a contrasting perspective, showing that high temperatures can hinder phenotypic evolution by destabilizing proteins and exacerbating the negative effects of neo-functionalizing mutations in cells. By examining the transition of a green fluorescent protein to a yellow fluorescent phenotype at different temperatures, the researchers were able to demonstrate that the rate of phenotypic evolution is significantly influenced by temperature. Specifically, the study showed that at high temperatures (44 °C), the excitation peak of the fluorescent protein shifted more slowly over 0–5 generations compared to lower temperatures. This suggests that the fluorescent protein evolves more slowly at high temperatures, likely due to the destabilizing effects of heat on proteins. At lower temperatures, protein folding stability is enhanced, which mitigates the destabilizing impact of neo-functionalizing mutations. This allows these mutations to confer a fitness advantage, boosting their proliferation and facilitating their rapid dissemination throughout the population (Zheng et al., 2024).

In the context of climate change, with increasing extreme weather and temperature fluctuations, the study of how microorganisms adapt to temperature stress becomes even more crucial. Microorganisms that are not naturally adapted to high temperatures (non-thermophilic) face significant challenges to their genomic stability, which can have far-reaching implications for their drug resistance, pathogenicity, and overall survival (Garcia-Solache & Casadevall, 2010; Huang et al., 2018; Nnadi & Carter, 2021). Understanding the mechanisms underlying temperature-adaptive phenotypes is a major research focus, as it can inform strategies to combat emerging infectious diseases and develop more effective drugs.

### 4.3 ROS

ROS, including superoxide radicals (O_2_^−^), H_2_O_2_, hydroxyl radicals (^•^OH), and singlet oxygen (^1^O_2_), can lead to multiple DNA damages, such as base excision, DNA-protein crosslinks, and single- or double-strand DNA breaks, without necessarily resulting in cell death (Rowe et al., 2008). Under normal growth conditions, the levels of intracellular ROS are kept low due to antioxidative systems, including small antioxidant molecules and enzymatic systems (Finkel & Holbrook, 2000). However, the ROS can be indirectly stimulated by environmental stressors such as high temperature, osmotic pressure, radiation, and numerous chemicals. Upon treatment with H_2_O_2_, *S. cerevisiae* exhibited significantly increased levels of recombination between homologs and LOH (Zhang et al., 2019). This study also confirmed that exposure to H_2_O_2_ can significantly elevate the rate of SNVs, up to two orders of magnitude, with A > T/T > A and C > G/G > C as the predominant base substitutions (Fig. 4c). These substitutions are likely indicative of the activity of DNA polymerase ζ (Northam et al., 2010). As mentioned above, intracellular ROS can be stimulated by various exogenous physical and chemical agents. Degtyareva et al. (2023) found that potassium bromate treatment produces a new type of ROS that cause frequent 8-oxo-G and G > T mutations (Fig. 4b). This raises the question: Would reducing ROS levels decrease genomic alterations in yeast? Zhang et al. (2019) observed that anaerobic incubation of yeast cells resulted in significantly reduced levels of recombination. However, the impact of anaerobic conditions on global genomic alterations in yeast remains to be explored.

### 4.4 Alcohols and aldehydes

Long-term experimental evolution (Voordeckers et al., 2015) and a *CAN1* mutation reporter assay (Voordeckers et al., 2020) have demonstrated that alcohol exposure moderately increases mutation rates. This mutagenic effect is attributed to the recruitment of error-prone polymerases to dysfunctional replication forks (Voordeckers et al., 2020). Aldehydes, belong to a group of chemicals referred to as reactive carbonyls, are widely present in our diets and the immediate environment. These compounds can induce DNA damage in cells by forming mutagenic DNA adducts, such as DNA breaks, base modifications, and cross-links (Vijayraghavan & Saini, 2023). In a yeast model, sequencing of *CAN1* mutants induced by formaldehyde and *lys2* frameshift revertants revealed frameshifts involving NER-dependent large deletions and complex insertions in hotspots of the *LYS2* gene (Grogan & Jinks-Robertson, 2012). This study highlighted that these complex mutations are mediated by NER as well as mutagenic bypass through polymerase ζ-mediated TLS. Another study demonstrated that formaldehyde, similar to acetaldehyde, is mutagenic to ssDNA in yeast, generating C > T and T > A transversions, a notable signature across many cancer types (Thapa et al., 2022). 5-Hydroxymethyl furfural (5-HMF), a prominent inhibitor found in baked foods and lignocellulosic hydrolysates during biofuel fermentation, is recognized for its potential antioxidant, anticarcinogenic, and anti-inflammatory properties (Choudhary et al., 2021). However, treatment of *S. cerevisiae* with 1.2 g/L 5-HMF disrupted intracellular redox status, reducing NADPH and glutathione levels, which in turn increased DNA recombination and chromosome aneuploidy, leading to increased DNA recombination and chromosome aneuploidy (Zhu et al., 2024). Although 5-HMF treatment had a minor impact on point mutations, it notably altered the base substitution pattern, especially reducing C > A/G > T substitutions. Whether this reduction reflects decreased formation of 8-oxo-G in 5-HMF-treated cells warrants further investigation (Zhu et al., 2024). Several studies have also shown that furfural, another furan-derived compound, triggers ROS in yeast cells (Allen et al., 2010). Qi et al. (2019) reported a 1.5- to 40-fold increase in the frequency of mitotic recombination in yeast exposed to furfural concentrations ranging from 0.1 g/L to 20 g/L. Their analysis of gene conversion tracts associated with crossovers revealed that furfural exposure induces DSBs during the G1 phase. Furfural-treated yeast exhibited a preference for C > A/G > T (Fig. 4b) and C > T/G > A transitions in SNVs (Qi et al., 2019; Qi et al., 2023b). This mutagenic effect was mitigated by BER pathway, as *ogg1* and *ung1* mutants displayed significantly higher mutation rates compared to wild-type strains under furfural treatment (Qi et al., 2023b).

### 4.5 Anti-tumor drugs

Anti-tumor drugs, such as methyl methanesulfonate (MMS), bleomycin, rapamycin, and cisplatin, are designed to target rapidly dividing cancer cells. MMS modifies DNA by adding methyl groups to various nucleophilic sites on the DNA bases, with 7-methylguanine (N7-MeG) and 3-methyladenine (N3-MeA) being the predominant adducts (Thompson & Cortez, 2020). These modified bases are particularly prone to depurination, leading to the formation of AP sites (Fig. 4d). The incorporation of adenine opposite AP sites by DNA polymerase δ frequently results in G > T and A > T base substitutions (Pagès et al., 2008). Nucleotide excision repair (NER) is the primary pathway for removing AP sites, and cells deficient in NER exhibit a significantly increased mutation frequency when exposed to MMS (Thompson & Cortez, 2020). Aside from N7-MeG and N3-MeA, MMS can also induce minor alkylation at other DNA sites, including the O6 position of guanine. The O6-methylguanine (O6-MeG) produced is particularly mutagenic because it preferentially pairs with T instead of C, leading to G > A transition mutations (Loechler et al., 1984; Wyatt & Pittman, 2006). When treated with 4 μg/mL Zeocin, a member of the bleomycin family, the rate of crossover and aneuploidy was increased by and 644- and 657-fold in a diploid *S. cerevisiae* strain (Sheng et al., 2019). Whole-genome sequencing of Zeocin-treated *S. cerevisiae* cells reveals a heightened rate of point mutations, including frequent T > G base substitutions and T deletions within 5′-GT-3′ sequences (Zheng et al., 2022). Fig. 4e illustrates that the error-prone DNA polymerase Rev1 can use AP sites as templates to incorporate cytosine (C) into the synthesis strand, converting it to guanine (G) in the next round of replication (Zheng et al., 2022). The T deletions observed at the 5′-GT-3′ motif are explained by DNA polymerase ζ-mediated template slippage following adenine insertion by Rev1. In contrast, rapamycin treatment reduces the mutation rate at the *CAN1* locus in yeast, suggesting a protective effect against nuclear DNA damage (Su et al., 2021). Nevertheless, rapamycin treatment is associated with the duplication of chromosome XII in yeast, compensating for ribosomal DNA contraction induced by the drug (Li et al., 2023). Cisplatin, a platinum-based anticancer drug, induces interstrand crosslinks between guanine residues (Dolling et al., 1999). The primary mutations induced by cisplatin at GC sites are base substitutions (C > T or C > A) or base deletions (loss of C), especially in yeast strains deficient in the NER protein Rad1 (Segovia et al., 2017). As depicted in Fig. 4f, cisplatin induces intra-strand crosslinks between two guanine bases, causing a break in one strand and the formation of a gap. During repair, DNA polymerase ζ may incorporate adenine opposite the crosslinked guanine or cause base deletion due to template slippage, leading to C > T mutations or single C deletions.

In addition to the stress factors mentioned above, various growth factors and other stressors can also induce genomic instability. For instance, Liu and Zhang (2019) demonstrated that yeast cells grown in a medium containing 1 M NaCl exhibited an 8-fold increase in InDel rate. Moreover, NaCl-treated cells showed a significantly higher ratio of small insertions to small deletions compared to untreated cells. The underlying mechanism driving this altered pattern, however, remains unclear. Since genomic variation arises from a combination of DNA damage and cellular repair mechanisms, understanding how different stress factors induce DNA damage and how cells utilize repair pathways under these conditions is crucial for comprehending the evolutionary dynamics of genomes in various environments.

## 5 Genomic alterations contribute to adaptive evolution in yeast

One positive outcome of studying genomic alterations in yeast is to guide the development of robust industrial strains. Under natural conditions, evolution occurs at a slow pace and is a long-term process. Selective pressure can accelerate the rate of genomic mutations, leading to the replacement of less-fit genotypes with more fit ones, thus driving adaptive evolution (Chen & Zhang, 2024; Conrad et al., 2011). For example, Cakar et al. (2005) developed a high-throughput procedure in 96-well plates combined with a most-probable-number assay to obtain strains with high resistance to freezing-thawing, temperature, ethanol, and oxidative stress under multiple selective pressures. Under the presence of an enzyme inhibitor terbinafine, adaptive evolution through serial subculture can yield yeast strains with higher squalene production efficiency. Comparative genomic analysis between the adaptive strains and the control strains identified F420I mutation in the *ERG1* gene improves squalene synthesis (Paramasivan et al., 2021). Chen and Zhang (2024) performed laboratory evolution of 3360 *S. cerevisiae* populations in 252 environments with varying levels of stress. They found that the adaptive genes mainly participate in signal transduction, transmembrane transport, or chemical homeostasis. Among them, the mutations on *IRA2* and *PDR1*, were further confirmed to improve the tolerance to certain stressors. This study systematically investigated the loci of adaptive evolution under a multitude of environmental factors, helping to uncover the genomic principles of environmental adaptation.

In addition to point mutations, large-scale chromosome rearrangements and aneuploidy also contribute to yeast adaptation under stressful conditions. For instance, the deletion of one copy of chromosome IX in diploid *S. cerevisiae* strains has been shown to enhance resistance to furfural (Qi et al., 2023b) and 5-HMF (Zhu et al., 2024). Inspired by these findings, researchers have devised methods to introduce large-scale genomic structural variations in yeast. Qi et al. (2023a) utilized CRISPR-Cas9 technology to create breaks in the conserved sequences of the transposon Ty1, stimulating extensive DNA recombination within the *S. cerevisiae* genome. Furthermore, the Synthetic Chromosome Rearrangement and Modification by LoxP-mediated Evolution (SCRaMbLE) system has emerged as a powerful tool for driving structural variations and even reshaping the yeast genome (Cheng et al., 2024; Steensels et al., 2018). Examples abound of how SCRaMbLE have produced evolved yeast strains with improved phenotypes. These include strains with increased tolerance to alkali (Ma et al., 2019), salt stress and 5-fluorocytosine (Kang et al., 2022), nocodazole (Cheng et al., 2024), as well as enhanced production of compounds such as deoxyviolacein and lycopene (Wang et al., 2018; Zhang et al., 2021). More examples detailing the pivotal role of structural variations in shaping industrially relevant traits and facilitating adaptive evolution have been comprehensively reviewed in Gorkovskiy and Verstrepen (2021) and Gorter de Vries et al. (2020). These insights not only deepen our understanding of the link between genomic alterations and adaptive advantages but also inform the rational design of genetic manipulation strategies aimed at tailoring yeast strains to meet specific industrial needs.

## 6 Conclusion

In this review, we emphasized how the integration of genetic screening systems with high-throughput sequencing in yeast models represents a powerful approach for studying induced genomic alterations and their broader implications for understanding genetic and environmental interactions. Our analysis revealed that various types of extracellular stressors cause different forms of damage to genomic DNA, driving the genome to evolve in distinct patterns. However, the global effects of significant environmental factors—such as temperature fluctuations, desiccation, pH changes, and shifts in carbon and nitrogen sources, which are common in both natural yeast habitats and industrial settings—are not yet fully understood. While specific environmental factors have been shown to induce characteristic genomic alterations, the mechanisms underlying these changes require further investigation. For example, LOH events have been observed to exhibit hotspots at the left end of chromosome VII under both spontaneous conditions and Zeocin treatment, but the reasons for this pattern remain unclear (Sui et al., 2020; Zheng et al., 2022). Furthermore, the current research results on yeast genomic mutation patterns are fragmented across different research groups. Integrating these data into a database and developing new data analysis tools would significantly advance this field. Additionally, while the budding yeast *S. cerevisiae* shares over 2000 genes (approximately 30% of its genome) with humans, significant differences exist in genome features and cellular mechanisms between the two organisms. Given that many conserved human genes can replace yeast genes and sustain yeast growth (Garge et al., 2020; Hamza et al., 2020; Laurent et al., 2020), creating 'humanized yeast'—yeast strains harboring human genomic DNA and proteins—could facilitate direct assays of human proteins involved in regulating genomic alterations within a simplified organismal context. Overall, as our understanding of the regulatory mechanisms governing genomic alterations improves in both natural and humanized yeasts, we anticipate gaining deeper insights into how exogenous factors intricately reshape the genome and impact health and disease outcomes.

## Conflicts of interest

The authors declare that there are no conflicts of interest.

## CRediT authorship contribution statement

**Ke-Jing Li:** Writing – review & editing, Writing – original draft, Methodology, Investigation, Data curation, Conceptualization. **Lei Qi:** Writing – original draft, Investigation. **Ying-Xuan Zhu:** Writing – review & editing, Writing – original draft, Conceptualization. **Min He:** Writing – original draft, Conceptualization. **Qian Xiang:** Conceptualization. **Dao-Qiong Zheng:** Writing – review & editing, Funding acquisition, Conceptualization.

## Declaration of competing interest

The authors declare that there are no conflicts of interest.
